# The LC-MS/MS Identification and Analgesic and Wound Healing Activities of *Lavandula officinalis* Chaix: In Vivo and In Silico Approaches

**DOI:** 10.3390/plants11233222

**Published:** 2022-11-24

**Authors:** Meryem Slighoua, Mohamed Chebaibi, Ismail Mahdi, Fatima Ez-zahra Amrati, Raffaele Conte, Mary Anne W. Cordero, Amal Alotaibi, Hamza Saghrouchni, Abdelkrim Agour, Touria Zair, Amina Bari, Dalila Bousta

**Affiliations:** 1 Laboratory of Biotechnology, Environment, Agro-Food and Health (LBEAS), Faculty of Sciences, Sidi Mohamed Ben Abdellah University, Fez 30050, Morocco; 2 Biomedical and Translational Research Laboratory, Faculty of Medicine and Pharmacy of the Fez, Sidi Mohamed Ben Abdellah University, B.P. 1893, Km 22, Road of Sidi Harazem, Fez 30000, Morocco; 3 AgroBioSciences Research Program, Mohammed VI Polytechnic University, Lot 660-Hay Moulay Rachid, Ben-Guerir 43150, Morocco; 4 Research Institute on Terrestrial Ecosystems (IRET)—CNR, Via Pietro Castellino 111, 80131 Naples, Italy; 5 Department of Basic Science, College of Medicine, Princess Nourah Bint Abdulrahman University, P.O. Box 84428, Riyadh 11671, Saudi Arabia; 6 Department of Biotechnology, Institute of Natural and Applied Sciences, Çukurova University, Balcali/Sariçam, Adana 01330, Turkey; 7 Laboratory of Natural Substances, Pharmacology, Environment, Modeling, Health and Quality of Life, Faculty of Sciences Dhar El Mahraz, Sidi Mohamed Ben Abdellah University, P.O. Box 1796, Fez 30000, Morocco; 8 Laboratory of Innovative Materials and Biotechnology of Natural Resources, Faculty of Sciences, Moulay 19 Ismail University, B.P. 11201, Meknes 50070, Morocco

**Keywords:** *L. officinalis* Chaix, LC/MS-MS, GC-MS, analgesic activity, wound-healing activity, molecular docking

## Abstract

We earlier emphasized in vivo the lavender plant’s (*Lavandula officinalis* Chaix.) anti-inflammatory and estrogenic activities and described the chemical compositions of its hydro-ethanolic (HE) extract. We used LC-MS/MS and GC-MS analyses to profile the phytochemical composition of the HE extract and to assess the analgesic and wound-healing effects of both the hydro-ethanolic (HE) and polyphenolic (LOP) extracts in vivo and in silico. The analgesic activity was studied using two methods: acetic acid and formalin injections in mice. The wound-healing activity was carried out over 25 days using a burn model in rats. In the in silico study, the polyphenols identified in the plant were docked in the active sites of three enzymes: casein kinase-1, cyclooxygenase-2, and glycogen synthase kinase-3β. The LC-MS/MS identified some phenolic compounds, mainly apigenin, catechin, and myricetin, and the GC-MS analysis revealed the presence of 19 volatile compounds with triazole, D-glucose, hydroxyphenyl, and D-Ribofuranose as the major compounds. The HE and LOP extracts showed significant decreases in abdominal writhes, and the higher licking time of the paw (57.67%) was observed using the LOP extract at 200 mg/kg. Moreover, both extracts showed high healing percentages, i.e., 99.31 and 92.88%, compared to the control groups, respectively. The molecular docking showed that myricetin, amentoflavone, apigenin, and catechin are the most active molecules against the three enzyme receptors. This study sheds light on the potential of *L. officinalis* Chaix as a source of natural products for pharmaceutical applications for analgesic purposes as well as their utility in promoting burn-healing activity.

## 1. Introduction

Aromatic and medicinal plants play a major role in the food, pharmaceutical, cosmeceutical, and perfume industries. *Lavandula officinalis* Chaix is a renowned multidisciplinary aromatic and medicinal plant [1]. This plant is used for plenty of pharmacological purposes, such as antispasmodic, relaxant, anti-inflammatory, carminative, and antidepressant properties. These beneficial effects are due to its richness in several aroma compounds and active substances, such as coumarins, sterols, and flavonoids [2].

Inflammation is the major defense mechanism of the body. It allows the immune machinery to recognize and eliminate harmful agents and proceed to the healing phase [3,4]. The inflammatory phase begins after an injury to immediately stop the bleeding by the hemostatic mechanism. The proliferative phase is characterized by the formation of a collagen bed and the production of new capillaries (granulation). Then, myofibroblasts decrease the size of the wound (wound contraction), and the proliferation of epithelial cells covering the new fabric (epithelialization) occurs. Finally, the phase takes place over several months (the remodeling phase), during which the dermis produces matrix proteins and collagen to regain its pre-injury phenotype. Several physiological events could affect the course of wound healing [5], among which the most important are the antioxidant, anti-inflammatory, analgesic, and antimicrobial activities [6].

Most injuries induce a feeling of pain, a sensation that causes patient discomfort. Pain is linked to the release of stress factors. It is an unwanted emotional or physical experience and could be acute or chronic. Although there are many drugs available for the treatment of chronic pain, they are challenged by several secondary effects, which present a major public health problem [7]. Following any injury, wound healing is the process of repairing damaged tissues. It is a dynamic and complex process of the replacement of the tissue layers and cellular structures. It involves an organized cascade of biochemical events divided into three main phases, the inflammatory, proliferative, and remodeling phases [8]. 

Previous studies have shown the relaxant effect of the flowers’ hydroethanolic extract of *L. officinalis* on tracheal smooth muscle. The possible mechanisms of action involve the blockage of muscarinic receptors, the inhibition of cyclooxygenase (COX) pathways, and the involvement of nitric oxide (NO) production [9]. Moreover, the ethanolic extract of the plant at the dose of 400 and 800 mg/kg has elicited anti-inflammatory activity using an in vivo model [10]. Indeed, Husseini et al. (2016) corroborated that *L. officinialis* flowers’ hydro-alcoholic extract was potent in inhibiting the pain induced by formalin and hot plate tests as well as inflammation using COX 1 and 2 activities in mice. Noteworthily, the administered extract resulted in comparable effects to those observed using morphine, dexamethasone, and indomethacin [11]. 

In wound healing, the Wnt/β-catenin signaling pathway plays a key role, which includes several proteins, such as glycogen synthase kinase-3β (GSK-3 β) and casein kinase-1 (CK1) [12]. Glycogen synthase kinase 3 β (GSK-3 β) is a key signaling pathway that may have a role in the control of wound healing. According to research by Zhang et al. (2008), the Wnt/b-catenin pathway in the Wnt signaling system could improve wound healing by inhibiting the GSK-3β protein. GSK-3β plays a variety of intricate roles in different cell and tissue types during the healing process of wounds [13,14]. Furthermore, a multifunctional protein kinase that is a member of the CK1 family of the serine/threonine kinases is called casein kinase-1 alpha (CK1). It participates in some signaling pathways, including the wound-healing process.

In the analgesic activity, the primary target of non-steroidal anti-inflammatory treatments is cycloxygenase (COX), the essential enzyme in prostaglandin (PG) formation, which regulates one of the fundamental routes of arachidonic acid metabolism. COX comes in two different isoforms, COX-1 and COX-2, with COX-2 playing a more significant role in inflammation, analgesic effects, and cell growth [15].

We previously demonstrated the anti-inflammatory and estrogenic properties of *L. officinalis* Chaix in vivo. To follow up on exploring the pharmacological potential of this plant, we have undertaken this study that aims to (1) define the phytochemical content of the HE extract of *L. officinalis* Chaix; (2) assess the analgesic and cutaneous burn-healing properties of its HE extract and the polyphenolic fraction; (3) investigate the in silico activity of the major secondary metabolites identified to obtain insight into their molecular mechanisms of action.

## 2. Results

### 2.1. Phytochemical Analysis with LC-MS/MS

**Figure 1:**
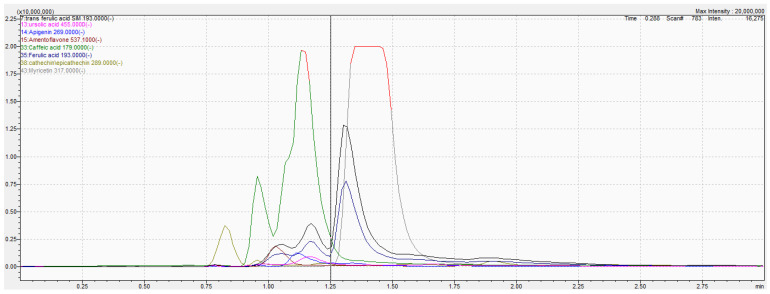
LC-MS/MS chromatogram of the hydro-ethanolic extract of *L. officinalis* Chaix.

The identification was achieved based on the molecular weight of the molecules in the ESI- mode. In particular, a selected ion monitoring (SIM) experiment was set, using the third quadruple of the MS/MS detector for acquisition. Eight compounds were found in the HE extract after the analysis, including trans-ferulic acid, ursolic acid, apigenin, amentoflavone, caffeic acid, ferulic acid, catechin, and myricetin, all belonging to the family of polyphenols (Figure 1, Table 1).

### 2.2. Phytochemical Analysis with GC-MS

**Figure 2:**
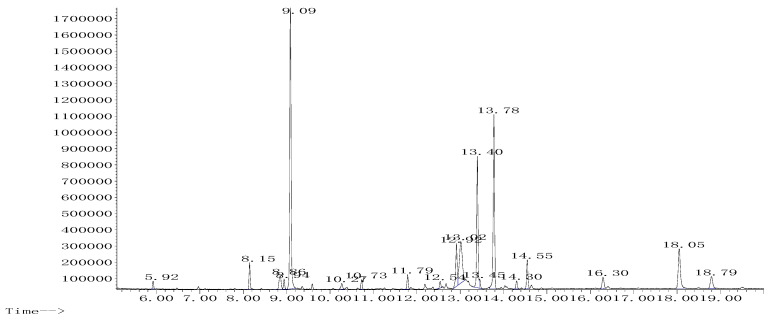
GC-MS chromatogram of the hydro-ethanolic extract of *L. officinalis* Chaix after silylation.

The GC-MS analysis of the HE extract showed the presence of nineteen compounds. According to surface area percentage, the most dominant compounds were triazole (29.31%), D-glucose (13.39%), hydroxyphenyl (18.21%), and D-Ribofuranose (9.56%) (Figure 2, Table 2).

### 2.3. Analgesic Activity

#### 2.3.1. Abdominal Writhes 

**Figure 3:**
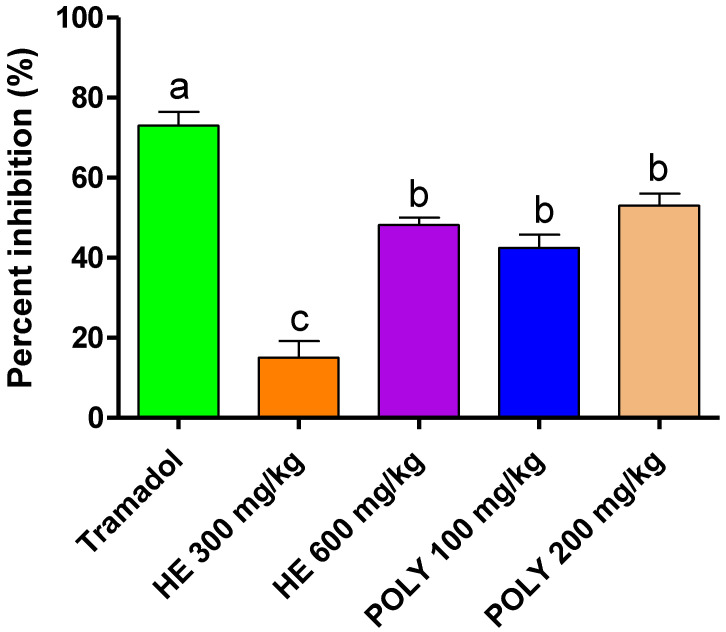
The inhibitory influence of *L. officinalis* Chaix. extracts on contortions in mice. The different letters in superscript (a,b,c) represent the treatment differences that are statistically significant at *p* < 0.05. Tramadol: reference analgesic drug.

Compared to the untreated rats, the HE extract at a dose of 600 mg/kg and the polyphenols at 100 and 200 mg/kg significantly reduced the abdominal contractions by up to 48.20%, 15.01%, and 53.33%, respectively (Table 3 and Figure 3). Considering this, the reference drug Tramadol was the most active in reducing writhing with significant variations compared to all of the treatments.

#### 2.3.2. Formalin-Induced Pain

**Figure 4:**
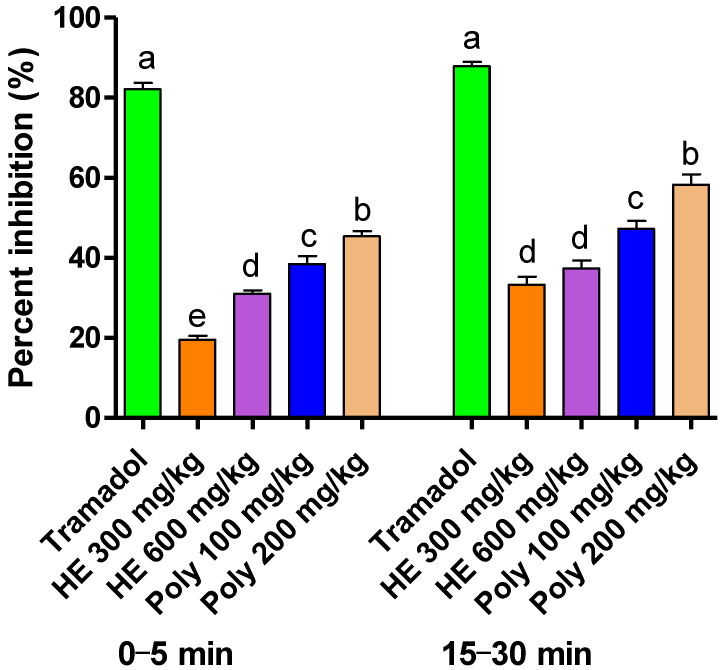
Percentage of inhibition of formalin-induced pain in mice upon the application of HE and polyphenolic extracts of *L. officinalis* Chaix and Tramadol (C+). The rates were calculated using the non-treated mice as a reference. The different letters in superscript (a,b,c,d) represent the treatment differences that are statistically significant *p* < 0.05.

At doses of 200 and 100 mg/kg, the polyphenolic extracts of *L. officinalis* Chaix showed more significant percentages of pain inhibition by 45.41% and 38.47% in the first phase and by 58.27% and 47.24% in the second phase, respectively (Table 4, Figure 4). The HE extract at a dose of 300 mg/kg and 600 mg/kg inhibited the pain by 19.52% and 31.02% in the first phase, respectively, and by 33.33% and 37.34% in the second phase, respectively. Noteworthily, Tramadol (the standard drug) was the most active in both phases, with up to an 85.7% reduction in the response time.

### 2.4. Wound-Healing Effect

**Figure 5:**
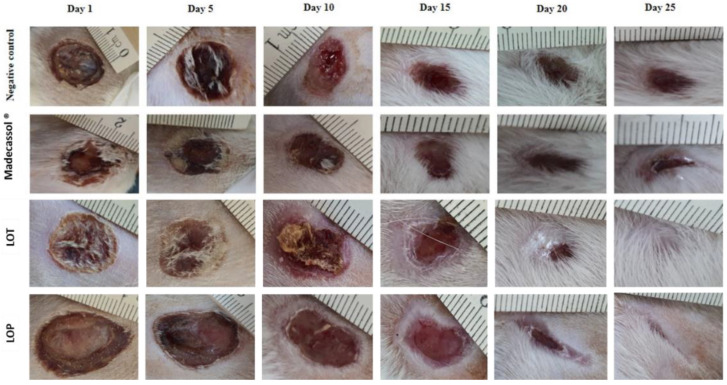
Photographic representation and morphological aspects of the burn-healing process using the hydro-ethanolic (LOT) and polyphenolic (LOP) extracts of *L. officinalis* Chaix and the control groups from day 1 to day 25.

Table 5 shows the effect of the topical application of the ointment prepared from the hydro-ethanolic and polyphenolic extracts of *L. officinalis* Chaix. A significant healing effect of the hydro-ethanolic extract was noted compared to the negative control. Figure 5 represents the progression of the healing process using the extracts and controls from day 1 to day 25. The wounds did not completely close in the negative control (Vaseline^®^), positive control (Madecassol^®^), and polyphenol-treated groups. However, the application of the hydro-ethanolic extract induced a complete cicatrization of the wound on the 25th day of treatment.

**Figure 6:**
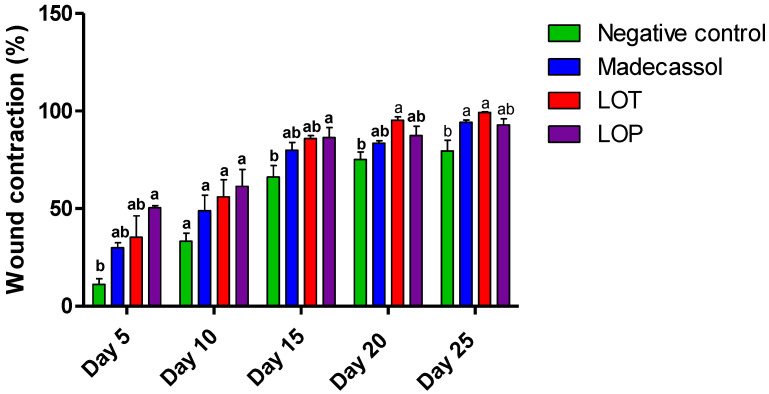
Wound-healing effect of the hydro-ethanolic (LOT) and polyphenolic (LOP) extracts of *L. officinalis* and the control groups. letters indicate significant differences (*p* < 0.05) using a one-way ANOVA analysis (Tukey’s test). The data represent the mean ± SD.

As shown in Figure 6, the polyphenolic extract of *L. officinalis* (50.44%) caused a strong contraction of the wound on the fifth day, followed by the hydro-ethanolic extract (47.06%). On day 25, the hydro-ethanolic extract of *L. officinalis* showed the highest healing percentage (99.31%), followed by the polyphenolic extract (92.88%) compared to the positive and negative control groups (94.27% and 80.62%, respectively).

### 2.5. Molecular Docking

The phytocompounds identified in the *Lavandula officinalis* hydro-ethanolic extract show important activity on casein kinase-1 (CK1) and glycogen synthase kinase-3β (GSK3B), two proteins. Myricetin, amentoflavone, and caffeic acid presented a glide score of −5.34, −5.186, and −4.897 Kcal/mol in the active site of casein kinase 1. Regarding the GSK-3β receptor, apigenin and catechin exhibited the highest glide score of −7.652 and −7.386 Kcal/mol in the active site of the GSK3-β receptor, respectively (Table 6). Then, we performed molecular docking to determine the score and glide energy of *L. officinalis* polyphenolic compounds with the cyclooxygenase-2 enzyme to assess their analgesic activity. Apigenin and catechin exhibited the highest glide score of −7.526 and −7.209 Kcal/mol in the active site of the cyclooxygenase-2 receptor (Table 6).

**Figure 7:**
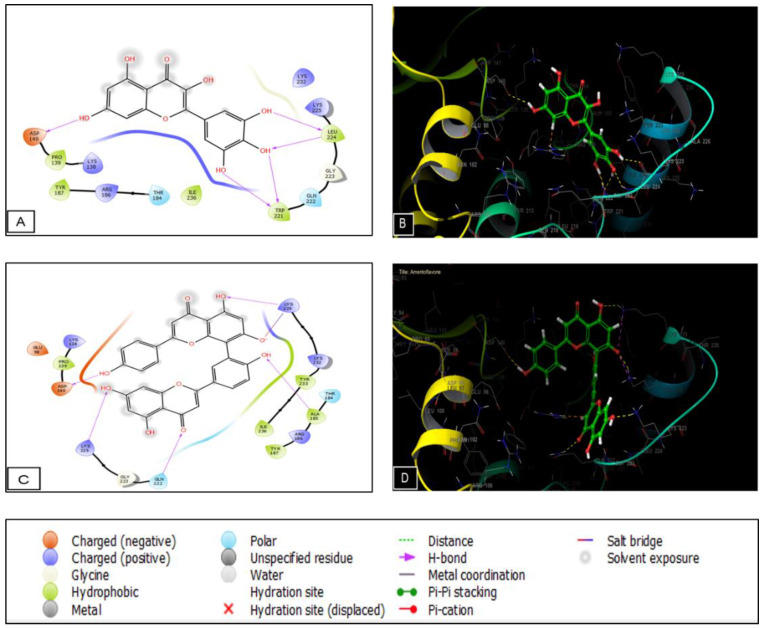
Myricetin interactions with CK1’s active sites are shown in (**A**) 2D and (**B**) 3D schematics. Diagrams of the mentoflavone interactions with the CK1 receptor’s active sites are shown in (**C**) 2D and (**D**) 3D.

**Figure 8:**
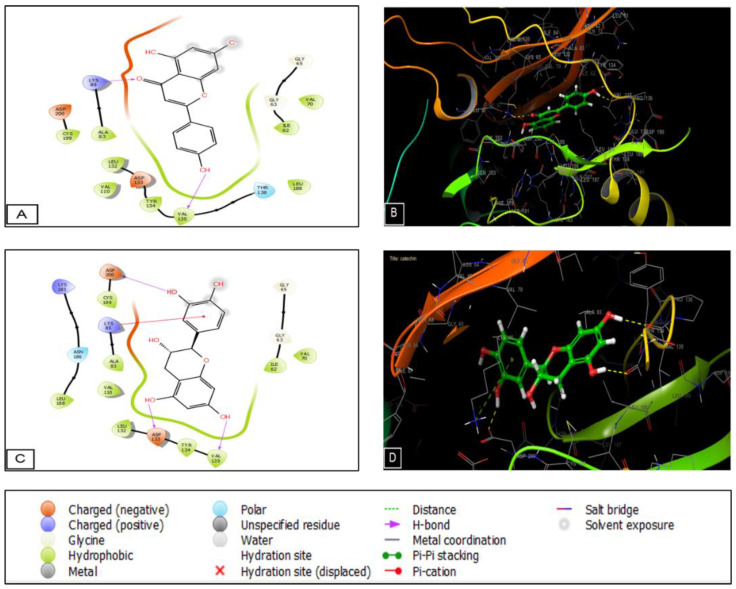
Diagrams showing the interactions between apigenin and the GSK3-β receptor’s active sites are shown in (**A**) 2D and (**B**) 3D. Diagrams of the catechin interactions with the GSK3-β receptor’s active sites are shown in (**C**) 2D and (**D**) 3D.

The numbers and types of potential bonds between the ligands in the active sites of CK-1 and GSK3-β are displayed in Figure 7 and Figure 8, respectively. In the CK-1 receptor, myricetin established five hydrogen bonds with residues ASP 140, TRP 221, and LEU 224. Amentoflavone established five hydrogen bonds with residues ASP 140, LYS 225, GLN 222, ALA 185, and LYS 229.

**Figure 9:**
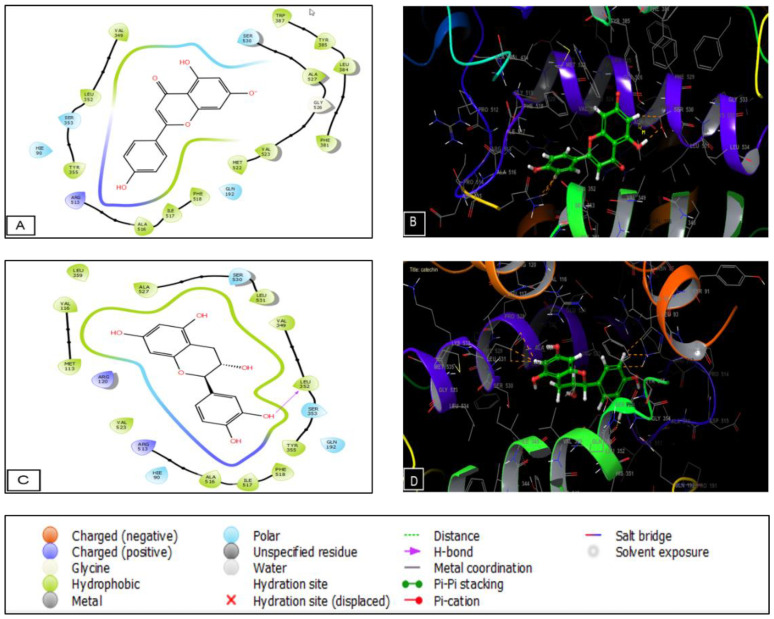
Diagrams showing apigenin’s interactions with the cyclooxygenase-2 receptor’s active sites are shown in (**A**) 2D and (**B**) 3D. Diagrams of the catechin interactions with the cyclooxygenase-2 receptor’s active sites are shown in (**C**) 2D and (**D**) 3D.

As for the GSK3-β receptor, apigenin established two H-bonds with LYS 85 and VAL 135. Moreover, catechin established three hydrogen bonds with ASP 200, ASP 133, and VAL 135 and one Pi-cation bond with LYS 85. Regarding the analgesic activity, catechin established one hydrogen bond with LEU 352 (Figure 9).

## 3. Discussion

Wound healing is one of the main physiological processes in the human body. It involves highly programmed steps, and many factors could interfere with normal healing [16]. In terms of treatments, topical therapies acting on targeted pathways have been primarily investigated as a promising strategy to enhance the healing process [17]. Among these, plenty of plants and derived phytochemicals were reported for their wound-healing potential in different in vivo models, including humans. However, the cutaneous wound-healing potential of most medicinal plants is still unexplored [18]. Hence, in this study, the in vivo wound-healing and analgesic properties of the polyphenolic fraction and the hydro-ethanolic extract of *L. officinalis* Chaix were evaluated. 

The phytochemical analysis of the *L. officinalis* Chaix plant with GC/MS revealed the presence of certain compounds similar to those identified in previous studies conducted on the phytochemistry of these plants [19,20,21]. Other compounds, such as sterols, triterpenes, and anthraquinones, were also identified [21,22,23]. These compounds are renowned for their pharmacological activities, such as D-glucose (anti-cancer and anti-diabetic activities) [24,25], hydroxyphenyl (reproduction and the endocrine system) [26], D-Ribofuranose (anti-inflammatory activity) [27] and myo-inositol (male and female fertility) [28,29]. Other studies revealed the presence of certain compounds in the hydro-ethanolic extract of lavender, such as malic acid [30], mannose and glucose [31], and cinnamic acid [32].

Apigenin, caffeic acid, ferulic acid, catechin, myricetin, and gallic acid have already been identified in *L. officinalis* Chaix [33,34,35]. Several previous studies have proven the antioxidant, anti-inflammatory, analgesic, and antibacterial effects of these compounds [36,37,38,39,40,41].

The results of the abdominal contortions suggest that this effect could be related to the mechanism of prostaglandin synthesis [42]. Indeed, acetic acid increases the release of prostaglandin (PGE2 and PGE2α) in the peritoneal fluid, partly involving the peritoneal receptors and inflammatory pain through the induction of capillary permeability (Ribeiro et al., 2000). That could also be due to the identified compounds which are known for their analgesic effects, such as palmitic acid, rosmarinic acid [43], kaempferol [44], arginine, apigenin [45], and myricetin [46].

Since nutrition is important for wound healing, many studies have shown that nutrient deficiencies are likely related to a delay in the wound-healing process. Several phytochemicals, such as polysaccharides, alkaloids, saponins, and phenolics, were shown to have healing properties [47,48], specifically palmitic acid [49], kaempferol [50], arginine [51], apigenin [52], and myricetin [53].

In the wound-healing process, casein kinase-1 (CK1) and glycogen synthase kinase-3β (GSK3B) are two proteins that play an important role in the activation of the Wnt/β-catenin signaling pathway and, therefore, the acceleration of wound healing [54]. In our in silico study, myricetin and amentoflavone were the most active molecules in the active sites of casein kinase-1, while apigenin and catechin were the most active in the active sites of glycogen synthase kinase-3β. Therefore, these molecules could activate the Wnt/β-catenin signaling pathway that leads to accelerated wound healing. The enzyme cyclooxygenase-2 is mainly responsible for acute pain, and cox-2 selective inhibitors are a better choice to stop the stimulatory action of pain [55]. Molecular docking showed that apigenin and catechin were the most active molecules in the active site of cyclooxygenase-2. In terms of analgesic activity, these two molecules could be natural inhibitors of cyclooxygenase-2 receptors.

## 4. Materials and Methods

### 4.1. Plant Material

The *L. officinalis* Chaix plant was grown in Imouzzer city, Meknes region, Morocco (33°440 N 5°010 W) [56]. The botanical identification was carried out by the botanist, Pr. Mohammed Ibn Tatou. A plant sample was deposited to the NHSI (National Herbarium and Scientific Institute) in Rabat, Morocco (voucher number RAB111499).

### 4.2. Animal Material 

Swiss albino mice (30 ± 4 g), having free access to water and food, were used in this study. The humidity was set at approximately 50% and a temperature of 22 °C, under a cycle of 12/12 h D/N. The manipulation of animals was carried out according to the EEC/86/EEC, the directive of the European community [57].

### 4.3. Preparation of the Extracts 

The lavender HE extract was prepared via the maceration of 10 g of the powder in 100 mL (70-30, *v*/*v*) of distilled water and ethanol at room temperature for 48 h. Using Whatman paper, the macerate was filtered, evaporated, and dried at 37 °C using a rotary evaporator [58].

The polyphenolic fraction was prepared by extracting 10 g of *L. officinalis* Chaix three times in 30 mL of methanol (50 °C, 3 h). Using a rotary evaporator, the solvent was then evaporated, dissolved in 50 mL of distilled water, and extracted 3 times in hexane (20 mL) and then in chloroform (in 20 mL). Afterward, the aqueous extract was extracted 3 times in ethyl acetate (20 mL), and under reduced pressure, the ethyl acetate was evaporated. In 30 mL of distilled water, the residue was redissolved and freeze-dried [59].

### 4.4. Phytochemical Analysis with LC-MS/MS

Ultra-high performance liquid chromatography (Shimadzu, Nexera XR, LC 40), coupled with a triple quadruple detector (LC/MS 8060, Shimadzu Italy, Milan), was used for the phytochemical analysis of the HE extract. The heating and nebulization gas flows were set at 10 L/min and 2.9 L/min, respectively. The drying gas flow was at 10 L/min, the DL temperature was at 250 °C, the heating block temperature was 400 °C, and the interface temperature was 300 °C. The internal database was developed, including polyphenol derivatives, through qualitative analysis. The separation of the compounds and standards was carried out on a C18 column, 3 × 100 mm, 2.6 μm (Phenomenex, Torrance, CA, USA). The mobile phase consisted of acetonitrile (A) and water +0.01% formic acid (B). The hydro-ethanolic extract of *L. officinalis* was added to acetonitrile and water (1:1). The solution (20 μL) was then diluted in acetonitrile (980 μL) and injected into the device for analysis [60].

### 4.5. GC-MS Analysis of the Phytochemicals 

To perform a phytochemical analysis, a dried extract of *L. officinalis* Chaix (1 mg) was dissolved in 100 mL of a HMDS-TMCS-Pyridine (3/1/9, (*v*/*v*/*v*)) reagent mixture. Then, the extract was incubated (30 min) and injected for analysis into the GC-MS, Agilent Technologies, Network 5973, MASS Selective detector with a capillary column (Agilent 19091S-433 model, Nominal length: 30 m, thickness: 0.25 μm, and diameter: 0.25 mm). Helium was used as carrier gas with a 31.4 mL/min total flow and split ratio set at 30:1. At 260 °C, the detector’s temperature was set. The temperature range for the 20-min run was between 60 and 300 °C. The injection was carried out in the splitless mode [61].

### 4.6. Analysis of the Activity

#### 4.6.1. Abdominal Writhes

Thirty mice were divided into six groups of five each. The first and second groups represented the negative (10 mL/kg NaCl) and positive controls (Tramadol 10 mg/mL). The HE extract of lavender was administered to the third and fourth groups at doses of 300 and 600 mg/kg, respectively. The polyphenolic fraction was issued to the fifth and sixth groups at doses of 100 and 200 mg/kg, respectively. After one hour, an intraperitoneal injection of acetic acid (1%) (10 mL/kg) was performed. The contortions were then counted after 10 min over a period of 20 min [62,63].

#### 4.6.2. Formalin-Induced Pain 

This test consisted of injecting formalin (10%) into the posterior paw of the mice after the oral treatment, 30 min beforehand, with the Tramadol and extracts. The time spent biting and licking the injecting paw was recorded using a stopwatch. The first phase was 0–5 min, and the second phase was 15–30 min. These two phases represented the sum of seconds spent biting and licking the paw after the formalin injection [64].

### 4.7. Wound-Healing Test 

#### 4.7.1. Preparation of the Ointments

The ointment was created by melting the extract (1 g) with Vaseline (9 g) using a water bath (50 °C) with a hustle. The ointments were then stored at 4 °C in airtight containers [65].

#### 4.7.2. Burn-Wound Induction 

In this test, 20 rats were used and divided into 4 groups of 5 rats each. The first and second groups represented the negative (Vaseline) and positive (1% Madecassol) controls, respectively. The third group was treated with the HE extract (10%), and the fourth group was treated with the polyphenolic extract (10%). The dorsal part of the rats was shaved (electric clipper) after anesthesia with pentobarbital (50 mg/kg). The burn was performed using a 1.7 cm aluminum rod heated for 10 s (110 °C). After 24 h, the ointments were applied to the burned area (for 25 days) by photographing it with a digital camera and using a ruler as a scale [66]. For the image analysis, ImageJ^®^ software was used. The following formula was used to measure the rate of wound contraction:

$$WC\;\left(\%\right)=\left[\frac{\left(WS0-WSSD\right)}{WS0}\right]\times 100$$

*WC* (%) = Wound contraction percentage
*WS*0 = Wound size on the first day
*WSSD* = Wound size at each specific day

### 4.8. Molecular Docking 

From the PubChem database, in SDF format, all phenolic chemicals discovered in the hydroethanolic extract of *L. officinalis* Chaix with LC/MS-MS were retrieved. Then, they were prepared for molecular docking with the OPLS3 force field using the LigPrep tool (Maestro 11.5 version of the Schrödinger Package software). For each ligand, thirty-two stereoisomers were produced after the ionization states at pH 7.0 ± 2.0.

The three-dimensional crystal structures in PDB format of casein kinase-1 (CK1) and glycogen synthase kinase-3 (GSK3-β) were downloaded from the protein data bank using the PDB IDs, 6GZD and 1Q5K, respectively, for the research of the healing activity. Additionally, the three-dimensional crystal structure of prostaglandin synthase-2 (cyclooxygenase 2) was retrieved in PDB format from the protein data bank under the PDB ID 6COX to explore the analgesic action. Schrödinger-Maestro v11.5’s Protein Preparation Wizard was used to create and polish the structures. All water molecules were eliminated, the heavy atoms were provided hydrogens, hydrogens were added to them, selenomethionines were converted to methionines, and the charges and bond orders were assigned. In order to minimize, the maximum heavy atom RMSD (root-mean-square-deviation) was set at 0.30 Å using the force field OPLS3.

By selecting any ligand atom, the receptor grid was automatically generated. The volumetric spacing performed was 20 × 20 × 20, and the coordinates were taken for the CK1 receptor as x: −7.55, y: −39.32, and z: −7.67; and for the GSK3-β receptor as x: 17.895, y: 20.948, and z: 11.786. As for cyclooxygenase 2, the coordinates were created using x: 70.401, y: 14.676, and z: 40.709.

Using Schrödinger-Maestro version 11.5, penalties were applied to non-cis/trans amide bonds during the standard precision flexible ligand docking. For the ligand atoms, the partial charge cutoff was set at 0.15, and the Van der Waals scaling factor and partial charge cutoff were set at 0.80. The final score was determined using the energy-efficient positions shown with the glide score. The lowest glide score value for each ligand’s best-docked position was noted [67].

### 4.9. Statistical Analysis 

A one-way ANOVA was used to analyze the results of each experiment, and the Tukey test was used for the post hoc analysis. The Tukey test was conducted in GraphPad Prism 6 software. The significance level was chosen at “*p* < 0.05”, and the values were presented as the mean ± S.D (standard deviation).

## 5. Conclusions

This study examined the hydro-ethanolic and polyphenolic extracts of *L. officinalis* Chaix’s phytochemical profile and evaluated the extracts’ in vivo analgesic and wound-healing effects. Our results confirmed some previous studies on the role of polyphenols as wound-healing and analgesic agents. However, the purification and assessment of pure compounds are necessary to prove their direct effect. It is also important to evaluate the physiological and molecular mechanisms underlying the healing and analgesic activities observed in vivo. Finally, this study highlighted the promoter and potential role of *L. officinalis* Chaix as a source of active phytochemicals suitable for developing plant-based healing and analgesic agents.

## Figures and Tables

**Table 1:** *L. officinalis* Chaix’s identifying composition with LC-MS/MS.

Molecules	Formula	Retention Time (min)	Molecular Adduct [M − H]^−^	Area under the Curve
Trans-ferulic acid	C_10_H_10_O_4_	1.311	193.00	60945002
Ursolic acid	C_30_H_48_O_3_	1.164	455.00	4652510
Apigenin	C_15_H_10_O_5_	1.116	269.00	3867802
Amentoflavone	C_30_H_18_O_10_	1.030	537.00	5516717
Caffeic acid	C_9_H_8_O_4_	1.141	179.00	115973902
Ferulic acid	C_10_H_10_O_4_	1.313	193.00	65420034
Catechin	C_15_H_14_O_6_	0.829	289.00	15052775
Myricetin	C_15_H_10_O_8_	1.430	317.00	254366279

**Table 2:** Compounds discovered in the *L. officinalis* Chaix HE extract using GC-MS.

Peak	Name	R.T (min)	Area %
1	Propanoic acid, 2-[(trimethylsilyl)oxy]	5.924	0.686
2	Cinnamic acid, o-methoxy-, trimethylsilyl ester	8.148	2.233
3	Benzoic acid trimethylsilyl ester	8.855	2.182
4	Anthracene, 5,6-dihydro	8.943	0.926
5	Triazolo[e]benzofurazan	9.088	29.319
6	Acrylic acid, 2-phenylethyl ester	10.268	0.786
7	Malic acid, tris(trimethylsilyl) ester	10.730	0.888
8	Glycolic acid-d2-o-(trimethylsilyl)	11.790	1.364
9	D-Glucitol, 1,1-di-C-octyl-2,3,4,6-tetra-O-trimethylsilyl	12.541	0.602
10	Arabinofuranose, 1,2,3,5-tetrakis-O-(trimethylsilyl)	12.914	4.598
11	D-Ribofuranose, 1,2,3,5-tetrakis-O-(trimethylsilyl)	13.015	9.566
12	D-Glucose, 2,3,4,5,6-pentakis-O-(trimethylsilyl)	13.396	13.396
13	Mannose, 2,3,4,5,6-pentakis-O-(trimethylsilyl)	13.453	0.791
14	P-hydroxyphenyl	13.778	18.219
15	Talose, 2,3,4,5,6-pentakis-O-(trimethylsilyl)	14.300	0.889
16	Synephrine (trimethylsilyl derivative)	14.549	2.976
17	Myo-Inositol, 1,2,3,4,5,6-hexakis-O-(trimethylsilyl)	16.295	1.690
18	Alpha-D-Galactofuranose, 1,2,3,5,6-pentakis-O-(trimethylsilyl)	18.050	6.678
19	Erythritol per-tms Butane, 1,2,3,4-tetrakis[(trimethylsilyl)oxy]	18.793	2.211
Total			100

**Table 3:** *L. officinalis* Chaix extracts’ impact on the mice’s response to acetic acid-induced writhing (*n* = 5).

Treatment	Dose (mg/kg)	Number of Writhes
Control		65.00 ± 2.88 ^a^
Tramadol	10	17.33 ± 1.45 ^d^
Hydro-ethanolic extract	300	55.00 ± 2.88 ^a^
	600	33.67 ± 1.85 ^bc^
Polyphenols	100	37.67 ± 2.18 ^bc^
	200	30.33 ± 0.66 ^c^

The different letters in superscript (a,b,c,d) represent the treatment differences that are statistically significant at *p* < 0.05. Control: mice treated with NaCl 0.9%, Tramadol: reference analgesic drug.

**Table 4:** Effect of the hydroethanolic and polyphenolic extracts of *L. officinalis* Chaix. on formalin-induced pain in mice.

Treatment	Dose (mg/kg)	Licking Time (s)	
		First Phase (0–5 min)	Second Phase (15–30 min)
Control		58.00 ± 0.50 ^a^	30.33 ± 2.50 ^a^
Tramadol	10	10.33 ± 0.80 ^d^	4.33 ± 1.70 ^d^
Hydro-ethanolic extract	300	46.67 ± 0.33 ^ab^	20.33 ± 0.66 ^b^
	600	40.00 ± 0.57 ^b^	19.00 ± 0.57 ^b^
Polyphenols	100	35.67 ± 0.88 ^b^	16.00 ± 0.57 ^bc^
	200	31.67 ± 0.88 ^bc^	12.67 ± 0.88 ^c^

The different letters in superscript (a,b,c,d) represent the treatment differences that are statistically significant at *p* < 0.05. Control: mice treated with NaCl 0.9%, Tramadol: reference analgesic drug.

**Table 5:** Wound size in cm^2^ after the treatment with the hydro-ethanolic and polyphenolic extracts of *L. officinalis* Chaix over 25 days.

Wound Size in cm^2^						
Treatments	Day 1	Day 5	Day 10	Day 15	Day 20	Day 25
Control	1.91 ± 0.15 ^a^	1.70 ± 0.18 ^a^	1.26 ± 0.03 ^a^	0.62 ± 0.05 ^a^	0.46 ± 0.03 ^a^	0.37 ± 0.06 ^a^
Madecassol^®^ (1%)	2.27 ± 0.12 ^a^	1.59 ± 0.09 ^a^	1.14 ± 0.11 ^a^	0.46 ± 0.10 ^a^	0.37 ± 0.04 ^a^	0.13 ± 0.03 ^b^
Hydro-ethanolic extract (10%)	2.83 ± 0.25 ^a^	1.22 ± 0.12 ^a^	1.01 ± 0.10 ^a^	0.33 ± 0.02 ^a^	0.10 ± 0.03 ^b^	0.01 ± 0.01 ^b^
Polyphénols (10%)	2.95 ± 0.28 ^a^	1.29 ± 0.25 ^a^	0.95 ± 0.11 ^a^	0.37 ± 0.14 ^a^	0.30 ± 0.10 ^a^	0.16 ± 0.06 ^ab^

Superscript letters indicate significant differences (*p* < 0.05) using a one-way ANOVA analysis (Tukey’s test). The data represent the mean ± SD. Madecassol: wound healing reference drug.

**Table 6:** Molecular docking with ligands in the CK1, GSK3-β, and cyclooxygenase-2 receptors.

	CK1 Receptor (PDB: 6GZD)		GSK3-β Receptor (PDB: 1Q5K)		Cyclooxygenase-2 Receptor (PDB: 6COX)	
	Glide Gscore (Kcal/mol)	Glide Energy (Kcal/mol)	Glide Gscore (Kcal/mol)	Glide Energy (Kcal/mol)	Glide Gscore (Kcal/mol)	Glide Energy (Kcal/mol)
Myricetin	−5.34	−37.995	−6.622	−42.144	−6.909	−28.13
Amentoflavone	−5.186	−43.924	−7.335	−52.265	-	-
Caffeic acid	−4.897	−25.316	−6.298	−27.62	−6.511	−29.447
Apigenin	−4.869	−29.071	−7.652	−36.061	−7.526	−34.259
Catechin	−4.62	−30.297	−7.386	−41.143	−7.209	−39.399
Ferulic acid	−4.596	−26.173	−6.669	−25.647	−6.156	−28.906

## Data Availability

All data included in the main text.
